# *VILMIR* is a *trans*-acting long noncoding RNA that enhances the host interferon response in human epithelial cells

**DOI:** 10.1128/jvi.01380-25

**Published:** 2025-12-11

**Authors:** Kristen John, Ethan Smith, Alexandra Istishin, Nasif Mahmood, Kayleigh Diveley, Tammy S. Tollison, Susan Carpenter, Xinxia Peng

**Affiliations:** 1 Department of Molecular Biomedical Sciences, North Carolina State University College of Veterinary Medicine 70727, Raleigh, North Carolina, USA; 2 Genetics & Genomics Graduate Program, North Carolina State University 6798 , Raleigh, North Carolina, USA; 3 Bioinformatics Graduate Program, North Carolina State University 6798 , Raleigh, North Carolina, USA; 4 Department of Molecular, Cell and Developmental Biology, University of California Santa Cruz 214546, Santa Cruz, California, USA; 5 Bioinformatics Research Center, North Carolina State University 6798 , Raleigh, North Carolina, USA; Loyola University Chicago - Health Sciences Campus, Maywood, Illinois, USA

**Keywords:** VILMIR, RNA-seq, ISG, lncRNA, interferon

## Abstract

Long noncoding RNAs (lncRNAs) have been found to play significant regulatory roles within antiviral and immune responses. We previously identified the novel lncRNA virus-inducible lncRNA modulator of interferon response (*VILMIR*), which was found to broadly regulate the host transcriptional response to interferon-beta (IFN-β) treatment in A549 human lung epithelial cells. Here, we investigated the mechanism by which *VILMIR* regulates the host interferon response in *trans* by identifying interacting proteins and gene regulatory networks of *VILMIR*. Through an RNA pull-down assay, we found that *VILMIR* interacted with both nuclear and cytoplasmic proteins *in vitro*, including the transcriptional regulators FUBP1 and PUF60 in the nucleus, as well as the antiviral proteins IFIT1 and IFIT3 and the aminoacyl-tRNA synthetases QARS1 and KARS1 in the cytoplasm. In addition, we found that the overexpression of *VILMIR* in A549 cells resulted in an overall enhancement of host interferon response genes and identified a core set of interferon-stimulated genes that were consistently regulated by *VILMIR* knockdown and overexpression. Finally, we proposed several possible mechanisms by which *VILMIR* may interact with the identified proteins to regulate the interferon response, such as by interacting with FUBP1 and PUF60 in the nucleus to regulate host transcription in *trans* or by interacting with the IFIT proteins and aminoacyl-tRNA synthetases in the cytoplasm to regulate translation.

**IMPORTANCE:**

Despite thousands of long noncoding RNAs (lncRNAs) being differentially expressed after immune responses and viral infections, there is limited knowledge on their individual functions in these contexts. We previously identified a novel lncRNA, *VILMIR*, that was found to be an interferon-stimulated gene that regulated the host transcriptional response to interferon-beta treatment in human epithelial cells. Here, we investigated the mechanism by which *VILMIR* regulates the interferon response. Through *in vitro* studies, we identified several nuclear and cytoplasmic proteins that interact with *VILMIR*, including proteins involved in transcriptional and translational regulation. In addition, we demonstrated that the overexpression of *VILMIR* results in an enhancement of host interferon response genes, supporting our hypothesis that *VILMIR* plays an activating role in the host interferon response. Finally, we propose several potential models for the mechanism of *VILMIR*, providing a foundation for the investigation of *VILMIR* as a novel therapeutic target in antiviral immunity.

## INTRODUCTION

Noncoding RNAs are known to be major regulators of cellular processes, such as ribosomal RNA (rRNA) and transfer RNA (tRNA), which are critical for protein synthesis (1, 2), as well as small nuclear RNA (snRNA) and small nucleolar RNA (snoRNA), which are critical for splicing and RNA modification (3, 4). The largest class of noncoding RNAs is long noncoding RNAs (lncRNAs), defined as transcripts greater than 500 nucleotides in length with low translational potential (5). The recent GENCODE V47 release estimates 35,934 lncRNA genes in the human genome (6). However, despite the large number of annotated lncRNAs, their functions are still widely unknown.

Individual lncRNAs have been identified to have significant functions within biological processes such as cell development (7), cancer (8), and inflammation (9) by regulating processes such as gene transcription and protein translation (10). There is also growing evidence that lncRNAs play important roles within antiviral and immune responses (11). For example, a recent study in 2023 identified that the overexpression of an lncRNA, LncRNA#61, inhibited influenza A virus (IAV) replication in human cells and even reduced viral replication *in vivo* after lipid nanoparticle-encapsulated delivery of LncRNA#61 in mice (12). As regulatory RNAs such as lncRNAs have low translational potential, they often function by interacting with RNA-binding proteins (RBPs) to regulate transcription (13) or act as scaffolds for protein interactions (14). While many lncRNAs have been found to act as *cis* regulators by regulating the expression of neighboring protein-coding genes (15), lncRNAs have also been found to function as *trans* regulators by regulating transcription on different chromosomes (16). Some lncRNAs can act in both *cis* and *trans*, such as *lincRNA-Cox2* that was found to regulate its neighboring gene, *Ptgs2*, as well as a subset of immune genes in *trans* using mouse models (17). In addition, a single lncRNA can have multiple functions in different cellular compartments, such as PYCARD-AS1 that facilitates DNA methylation at the *PYCARD* promoter in the nucleus, as well as interacts with PYCARD mRNA in the cytoplasm to inhibit ribosome assembly (18). Therefore, understanding the RBP interactions of an lncRNA can help elucidate how it functions within the cell.

We previously identified a novel lncRNA named virus-inducible lncRNA modulator of interferon response (*VILMIR*) that was found to regulate the host transcriptional response to both interferon-beta (IFN-β) treatment and IAV infection in A549 human lung epithelial cells (19). We found that *VILMIR* did not regulate transcription of its neighboring protein-coding genes but rather had a broad transcriptional regulation; however, the exact mechanism of this regulation was not explored. Therefore, in this study, we aimed to identify interacting proteins and gene regulatory networks of *VILMIR* to better understand its molecular interactions and how it regulates the interferon response in *trans*. Using an RNA-pull down assay, we found that *VILMIR* interacts with several proteins in A549 nuclear and cytoplasmic lysates, including the transcriptional regulators FUBP1 and PUF60, as well as antiviral proteins IFIT1 and IFIT3 and aminoacyl-tRNA synthetases (ARSs) QARS1 and KARS1. In addition, we overexpressed *VILMIR* in A549 cells and found that the overexpression resulted in an overall enhancement of host interferon response genes, supporting our hypothesis that *VILMIR* plays an activating role in the host interferon response. By combining RNA-seq analyses from both *VILMIR* knockdown (KD) and overexpression studies, we further identified a core set of genes that are consistently regulated by *VILMIR* perturbation in *trans*. Finally, we proposed several potential models of *VILMIR* function, suggesting that *VILMIR* may function in both the nucleus and the cytoplasm to regulate host interferon responses.

## MATERIALS AND METHODS

### Cell culture

The human cancer cell line, A549 lung epithelial (CCL-185), was purchased from American Type Culture Collection (ATCC, Manassas, VA, USA). Human embryonic kidney (HEK) epithelial 293 FT cells were ordered from Invitrogen (ThermoFisher Scientific). A549 cells were maintained in F-12K media with 10% fetal bovine serum (FBS). HEK 293 FT cells were maintained in DMEM media plus 1% GlutaMAX and 10% FBS. All cell lines were kept at 37°C in a 5% CO_2_ incubator and maintained in culture, as recommended by the ATCC.

### Protein extraction

In order to collect protein lysates for subsequent RNA pull-down assay, confluent T182 flasks of A549 cells were washed with 1X Dulbecco’s phosphate-buffered saline and treated with fresh A549 media containing 1 ng/mL human IFN-β recombinant protein (R&D Systems 8499IF010) for 6 hours. Nuclear and cytoplasmic protein lysates of the cells were extracted using NE-PER Nuclear and Cytoplasmic Extraction Reagents (Thermo Scientific) according to the manufacturer’s instructions. Relative protein quantification was determined using absorbance at 280 nm by comparison with a bovine serum albumin (BSA) standard curve.

### RNA pull-down assay and mass spectrometry

Full-length *VILMIR* (Table S1) or its antisense sequence was cloned into the pGEM-3Z vector (Promega) downstream of the T7 promoter using EcoRI and BamHI restriction enzyme sites. The plasmids were linearized with BamHI on the 3′ end in order to facilitate *in vitro* transcription. Biotinylated RNA probes were *in vitro-*transcribed by the T7 RNA polymerase using the TranscriptAid T7 High Yield Transcription Kit (Thermo Scientific) and a 1:39 molar ratio of Biotin-16-UTP (ApexBio Technology) to standard UTP. Subsequent RNA was purified using the RNeasy Plus Mini Kit (Qiagen) with a genomic DNA eliminator column, and the size was determined with an Agilent Bioanalyzer or TapeStation 4150.

The pull-down assay was adapted for RNA from Springer Protocols (20). Briefly, 1.5 mg of prewashed Dynabeads M-280 Streptavidin (Invitrogen) was incubated with 25 µg of *VILMIR* sense or antisense RNA probes at room temperature with agitation for 30 min. Excess biotinylated RNA probe was removed from the bead-probe complex by several washes. Approximately 3.6 mg of nuclear or 5.3 mg of cytoplasmic protein lysate from A549 cells was incubated with 12.5 µg of antisense bead:probe complexes at 4°C, with agitation for 30 min as a preclearing step. Precleared lysates were then incubated with *VILMIR* sense or antisense bead:probe complexes with 50 µg tRNA competitor (Thermo Scientific) and 300 U of SUPERase·In RNase Inhibitor (Invitrogen) at 4°C with agitation for 1 h. Bead:probe:protein complexes were washed three times in washing buffer, eluted in water at 70°C for 5 min, and boiled in Laemmli buffer at 95°C for 10 min. The pull-down assay was performed in triplicate for both sense and antisense reactions.

The supernatant was run on a 6% SDS-PAGE gel for approximately 10 min, and then the gel was stained with Coomassie blue and destained to excise and prepare the samples for protein identification by mass spectrometry. Mass spectrometry was performed by the BIDMC-Harvard Mass Spectrometry Facility and Asara Laboratory using the Thermo Scientific QExactive HFx Orbitrap nano HR-LC-MS/MS following in-gel digestion of proteins with Trypsin/LysC.

To identify *VILMIR* sense-specific binding proteins in the nucleus or cytoplasm, we first averaged the peptide spectrum counts in the sense and antisense replicates and then calculated an approximate fold-change (FC) value by taking the difference between the sense and antisense peptide counts (sense/antisense). Proteins that were evenly distributed between the sense and antisense samples (i.e., FC of 1), enriched in the antisense samples (FC <1), or whose replicates had inconsistent counts were eliminated. *VILMIR* sense-specific bound proteins were identified as those proteins that had protein peptide spectrum counts in all three replicates of the sense RNA but none in the antisense replicates (unique binding), or whose peptide spectrum counts were enriched (FC >1) in the sense replicates over the antisense replicates (enriched binding). These remaining proteins were narrowed down further by removing proteins with an FC <2 in the sense replicates, as well as focusing on proteins with known associations with interferon and antiviral responses.

### Western blotting

Identified proteins from mass spectrometry were confirmed by Western blot after a pull-down assay, as described above. The starting protein lysate for *VILMIR* sense and antisense pull-down assay was standardized before pull-down. After pull-down, an equal volume of the eluted protein from the sense and antisense pull-down was loaded into 10% SDS-PAGE gels and transferred to polyvinylidene difluoride (PVDF) membranes (Invitrogen). Membranes were blocked for either 1 h at room temperature or 4°C overnight in 1× Tris-Buffered Saline (TBS) with 1% (wt/vol) casein and then incubated in primary antibody diluted in blocking buffer for 1 h at room temperature. The following primary antibodies were used: anti-FUBP1 1:1,000 (Proteintech, 24864-1-AP), anti-PUF60 1:1,000 (Proteintech 10810-1-AP), anti-PCNA 1:5,000 (Proteintech, 10205-2-AP), anti-IFIT1 1:500 (Cell Signaling Technology, #14769), anti-IFIT3 1:2,000 (Proteintech, 15201-1-AP), anti-GlnRS (or QARS1) 1:2,000 (Proteintech 12645-1-AP), anti-KARS 1:1,000 (Proteintech 14951-1-AP), and anti-GAPDH 1:5,000 (Proteintech, 10494-1-AP). Membranes were rinsed 2× and washed 2× for 5 min each in TBS buffer with 0.05% Tween 20 (TBS-T) and then incubated in Goat anti-Rabbit IgG (H + L) secondary antibody, HRP conjugate (Invitrogen #31460) diluted 1:5,000 in blocking buffer for 30 min at room temperature. Membranes were rinsed 3× and washed 3× for 5 min each in TBS-T buffer and then detected by chemiluminescence using either Pierce ECL Substrate (Thermo Scientific) for the FUBP1 and PCNA blots or SuperSignal West Atto Ultimate Sensitivity Substrate (Thermo Scientific) for the remaining blots, according to the detection limits of the protein. The blots were visualized using a Bio-Rad ChemiDoc MP Imaging System. Densitometry analysis was performed using ImageJ software where applicable. Briefly, the background was subtracted, and the density of each band was measured. In order to estimate a relative FC between the sense and antisense protein interactions, the sense and antisense bands were then normalized to their input band, and the average FC of three independent replicates was calculated. With FUBP1, PUF60, and QARS1 that displayed several bands, all bands were included so as to not bias the analysis. FUBP1 and PUF60 were not included in the densitometry analysis as the antisense band was too low to obtain an accurate density.

### Interferon treatment

Following the same methods as 19, A549 cells were seeded overnight between 150,000 and 175,000 cells per well in 12-well plates in 1.5 mL media. The following day, the cell monolayer was washed with 1× DBS and treated with fresh A549 media with or without human IFN-β recombinant protein at the indicated concentrations. All cells were harvested at 6 hours after treatment according to the TRIzol Reagent User Guide (Invitrogen).

### RNA isolation and quantitative PCR

Total RNA was isolated from cells following the TRIzol isolation method (Invitrogen) and quantified using NanoDrop spectrophotometry. One microgram of RNA was reverse-transcribed into cDNA using the QuantiTect Reverse Transcription Kit (Qiagen) containing both oligo-dT and random primers. Quantitative PCR (qPCR) was performed on the cDNA using PowerUp SYBR Green Master Mix (Applied Biosystems). Relative expression of the indicated RNAs was determined using the ΔΔCt method with GAPDH as an endogenous control. Statistical analysis of significance was performed in JMP Pro 16 software (SAS Institute Inc., Cary, NC). The primer sequences used in this study are as follows: *GAPDH* F: GGTATCGTGGAAGGACTCATGAC; *GAPDH* R: ATGCCAGTGAGCTTCCCGTTCAG (21); *VILMIR* F: GCTCCACCCTGAAAGTC; *VILMIR* R: CTACACAGTGCTGAGGAAA (19).

### Plasmid construction and overexpression of *VILMIR*

The pSico bidirectional expression vector was a gift from Susan Carpenter and described in 22. Full-length *VILMIR* was cloned into the pSico vector with an EF1a promoter expressing zeocin resistance and GFP as a selection marker, using PspXI and NotI restriction enzyme sites. The sequence was confirmed by Sanger sequencing. To produce lentivirus, HEK-293FT cells were co-transfected with the pSico vector expressing *VILMIR* and lentiviral vectors, psPAX2 (Addgene #12260) and pMD2.G (Addgene #12259) using the Lipofectamine 3000 Reagent (Invitrogen). In addition, an empty pSico vector was transfected as a negative control. The viral supernatant was collected 72 h post-transfection and filtered through a 0.22 µM syringe filter. A549 cells were transduced with the lentivirus, and stable integrants were sorted based on GFP expression at the UNC Flow Cytometry Core Facility (Chapel Hill, North Carolina) using a Becton Dickinson FACSAria II. The successful overexpression of *VILMIR* was confirmed by RT-qPCR.

### cDNA library construction, RNA-sequencing, and Ingenuity Pathway Analysis (IPA)

mRNA sequencing was performed in biological triplicate in A549 *VILMIR*-overexpressing and control cell lines treated with mock or either 1 ng/mL or 10 ng/mL human IFN-β for 6 h. Total RNA was isolated from cells following the TRIzol isolation method (Invitrogen). All samples were quantified and assayed to confirm a minimum RNA integrity number of at least 9.7 using an Agilent TapeStation 4150. Next, 500 ng of total RNA per sample underwent mRNA capture and was then fragmented at 94°C for 6 min. Sequencing libraries were prepared according to the manufacturer’s protocol using 11 cycles of final amplification (KAPA mRNA HyperPrep Kit, catalog no. KK8580 and KAPA UDI Adapter Kit, catalog no. KK8727). Libraries underwent QC prior to sequencing using an Agilent TapeStation 4150. Next-generation sequencing was performed on a Complete Genomics DNBSEQ-G400C (150 bp paired end) to a targeted depth of ~20 million reads per sample. The sequencing data from *VILMIR* KD in 19 were also incorporated.

Complete genomics RNA-seq reads were mapped against the Hg38 using STAR version 2.7.9 a (23). Custom STAR parameters were set as follows: limitOutSAMoneReadBytes: 1,000,000, outSAMprimaryFlag: AllBestScore, outFilterType: BySJout, alignSJoverhangMin: 8, alignSJDBoverhangMin: 3, outFilterMismatchNmax: 999, alignIntronMin: 20, alignIntronMax: 1,000,000, alignMatesGapMax: 1,000,000, and outFilterMultimapNmax: 20; otherwise, default STAR parameters were used. Following read mapping, a count matrix was generated from the STAR results using R. Genes were removed from the matrix if they did not have at least 30 reads in a minimum of three samples from either the perturbation group (KD or overexpression) or the control group. Counts were normalized using the TMM normalization method via the calcNormFactors function in edgeR version 3.40.2 (24).

To conduct our differential gene expression analysis, we utilized the limma-trend approach from Limma version 3.54.2 (25). For each dose level of the IFN-β treatment, we assessed differential expression by comparing each KD or overexpression condition to its respective mock treatment. We then contrasted the differential expression results of each KD or overexpression (STAT1g1/1 ng IFN-β vs STAT1g1/Mock, VILMIRg1/1 ng IFN-β vs VILMIRg1/Mock, etc.) against that of its corresponding control (Ctrl/1 ng IFN-β vs Ctrl/Mock, Ctrl/10 ng IFN-β vs Ctrl/Mock). Genes were considered differentially expressed in a given contrast if their unadjusted *P*-value was less than 0.05 with no FC requirement. To identify IFN-responsive genes, we further filtered these results by requiring a log2FC greater than 1.25 following IFN-β treatment in the control cell lines for each experiment. Differential expression results were visualized using the ComplexHeatmap R package version 2.14.0 (26).

Pathway enrichment analysis was generated using QIAGEN Ingenuity Pathway Analysis (IPA) (27). A raw *P*-value cutoff of <0.05 was used to define genes with significant expression changes after *VILMIR* overexpression in each IFN-β treatment. Canonical pathways analysis identified the pathways from the QIAGEN IPA library of canonical pathways that were most significant to the data set. Differentially expressed genes from the data set that met the *P*-value cutoff of 0.05 (−log10 *P*-value 1.3) and were associated with a canonical pathway in the QIAGEN Knowledge Base were considered for the pathway analysis. A right-tailed Fisher’s exact test was used to calculate a *P*-value determining the probability that the association between the genes in the data set and the canonical pathways is explained by chance alone.

## RESULTS

### LncRNA *VILMIR* can interact with nuclear and cytoplasmic proteins from A549 epithelial cells *in vitro*, including FUBP1, PUF60, IFIT1, IFIT3, QARS1, and KARS1

To identify potential protein interactions of *VILMIR*, we performed an RNA pull-down assay. As *VILMIR* was localized in both nuclear and cytoplasmic compartments in A549 human lung epithelial cells (19), we investigated its potential protein interactions in both compartments. *In vitro-*transcribed biotinylated *VILMIR* as well as antisense *VILMIR* control RNA were incubated with nuclear or cytoplasmic lysates of A549 cells treated with IFN-β to mimic cellular interactions during a host interferon response. Interacting RBPs were then identified by mass spectrometry. The full list of identified proteins was narrowed down by prioritizing proteins that either uniquely interacted with *VILMIR* sense RNA compared to antisense RNA or were greater than two times enriched in the *VILMIR* sense RNA compared to antisense RNA according to peptide spectrum counts (see Materials and Methods). From these criteria, we identified 19 proteins in each compartment that were either unique to or enriched in the *VILMIR* sense RNA (Tables 1 and 2). This suggests that *VILMIR* may have functions in both compartments.

**TABLE 1:** Mass spectrometry identification of *VILMIR*-interacting proteins in A549 nuclear cellular lysate treated with IFN-β, as determined by RNA pull-down assay

Protein ID	Mass (kDa)	Sense *VILMIR* peptide count			Antisense *VILMIR* peptide count			Enrichment (avg sense/avg antisense)
LRPPRC	158	19	15	15	0	0	0	N/A^*a*^
PCBP2	39	10	8	2	0	0	0	N/A
CSTF3	83	10	9	10	0	0	0	N/A
CELF1	52	11	9	7	0	0	0	N/A
CSTF2	61	6	5	5	0	0	0	N/A
TARDBP	45	6	5	3	0	0	0	N/A
CPSF7	52	5	4	4	0	0	0	N/A
FUBP1	68	24	24	23	5	2	2	7.9
KHSRP	73	87	91	84	15	12	9	7.3
FUBP3	62	8	7	5	0	0	1	6.7
ELAVL1	36	6	3	3	0	0	2	6.0
CSTF1	48	122	133	135	27	26	18	5.5
SYMPK	141	4	7	5	1	0	0	5.3
SF1	68	54	61	65	14	15	14	4.2
HNRNPM	78	20	23	19	6	5	4	4.1
PUF60	60	46	55	52	16	18	15	3.1
CPSF1	161	29	28	35	10	10	10	3.1
CPSF2	88	39	40	38	15	14	13	2.8
SCAF11	165	85	89	72	36	37	33	2.3

^*a*^ N/A, not applicable, as all peptide counts in the antisense were zero.

**TABLE 2:** Mass spectrometry identification of *VILMIR*-interacting proteins in A549 cytoplasmic cellular lysates treated with IFN-β, as determined by RNA pull-down assay

Protein ID	Mass (kDa)	Sense *VILMIR* peptide count			Antisense *VILMIR* peptide count			Enrichment (avg sense/avg antisense)
IFIT3	56	4	7	4	0	0	0	N/A*^a^*
LARS1	134	16	16	9	1	0	0	13.7
IFIT1	55	10	12	11	1	1	1	11.0
QARS1	88	15	14	14	2	1	2	8.6
AIMP1	34	8	9	5	1	0	1	7.3
MARS1	101	17	16	10	2	2	3	6.1
HNRNPK	51	22	22	17	5	5	3	4.7
RARS1	75	23	13	15	3	3	5	4.6
KARS1	68	48	35	45	12	9	9	4.3
PCBP2	39	4	3	4	0	1	0	3.7
FUBP1	68	31	38	37	12	10	11	3.2
IARS1	145	36	40	34	13	10	13	3.1
SART3	110	12	11	7	4	3	3	3.0
PUF60	60	16	22	20	7	6	10	2.5
U2AF2	54	13	11	11	6	6	2	2.5
DARS1	57	21	19	20	9	7	10	2.3
LRPPRC	158	138	141	141	68	62	55	2.3
EPRS1	171	35	35	18	12	14	13	2.3
SF3B3	136	8	11	7	3	4	5	2.2

^*a*^ N/A, not applicable, as all peptide counts in the antisense were zero.

**Fig 1:**
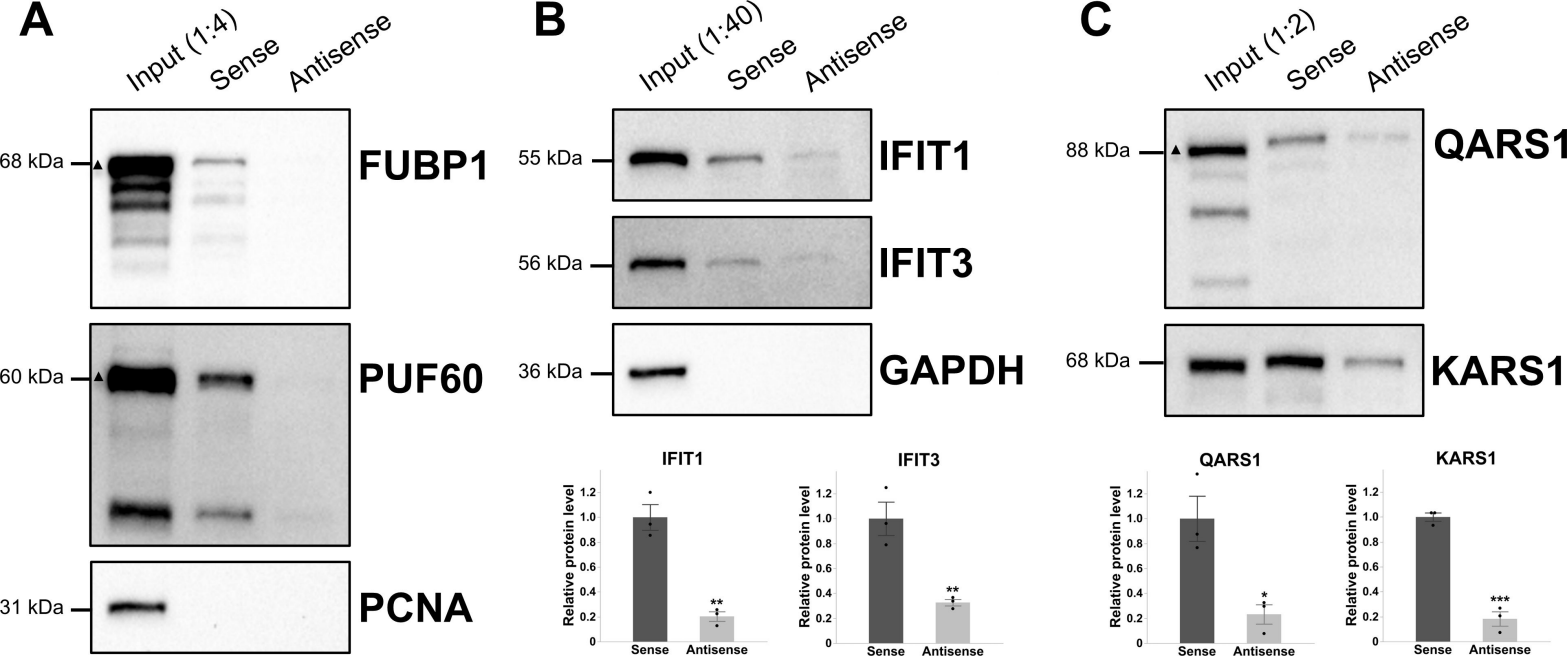
*VILMIR* sense RNA interacts with nuclear and cytoplasmic proteins from the A549 lysate. An RNA pull-down assay was performed by incubating *in vitro-*transcribed *VILMIR* sense RNA or antisense RNA (negative control) with nuclear or cytoplasmic lysates from IFN-treated A549 cells. Interacting proteins were identified by mass spectrometry. The full list of identified proteins can be found in Tables 1 and 2. (**A**) The interaction of *VILMIR* sense RNA with FUBP1 and PUF60 was confirmed by Western blot. PCNA was used as a negative nuclear control, and the input protein was diluted 1:4. (**B**) The interaction of *VILMIR* sense RNA with IFIT1 and IFIT3 was confirmed by Western blot with GAPDH as a negative cytoplasmic control, and the input protein was diluted 1:40. Densitometry analysis of IFIT1 and IFIT3 protein bands in the Western blots is displayed below the blots. (**C**) The interaction of *VILMIR* sense RNA with QARS1 and KARS1 was confirmed by Western blot, and the input protein was diluted 1:2. Densitometric analysis of QARS1 and KARS1 protein bands in the Western blots is displayed below the blots. All Western blots are representative of three independent replicates. An equal volume of the sense and antisense pull-down protein was loaded into the gel, and the input lane was included to estimate a relative FC between sense and antisense protein interaction. The labeled size in kilodaltons (kDa) denotes the major band for each protein, as well as a triangle for proteins that display multiple bands. FUBP1 has known shorter isoforms (30), while the additional bands for PUF60 and QARS1 are likely background bands. All bands were included so as not to bias the analysis. Proteins were detected by chemiluminescence using either Pierce ECL Substrate (Thermo Scientific) for the FUBP1 and PCNA blots (**A**) or SuperSignal West Atto Ultimate Sensitivity Substrate (Thermo Scientific) for the remaining blots (PUF60 in A and B–C). Densitometry analysis was not performed for panel A as the antisense band was too low to obtain an accurate density. *P < 0.05, **P < 0.01, and ***P < 0.001 (Student’s *t*-test).

In the nuclear A549 lysate, *VILMIR* sense RNA was found to interact with several proteins involved in pre-mRNA splicing and processing (CSTF1, CSTF2, CSTF3, CELF1, CPSF7, SYMPK, SF1, HNRNPM, CPSF1, CPSF2, and SCAF11), as well as transcriptional regulation (TARDBP, FUBP1, KHSRP, FUBP3, and PUF60). Using Western blot analysis, we confirmed the interaction of *VILMIR* sense RNA with FUBP1 and PUF60 (Fig. 1A). FUBP1, or Far Upstream Element-Binding Protein 1, acts as a transcriptional regulator and is a well-known activator of the *c-Myc* oncogene (28). While FUBP1 is primarily located in the nucleus, it has been found to translocate to the cytoplasm (29) and was also identified in our cytoplasmic mass spectrometry results (Table 2). To negatively control the expression of *c-Myc*, FUBP1 also interacts with the FUBP-Interacting Repressor (FIR), which is an alternatively spliced variant of Poly(U)-Binding Splicing Factor 60 (PUF60), meaning that FUBP1 can be involved in both positive and negative regulation of gene expressions (28).

Interestingly, in the cytoplasmic A549 lysate, *VILMIR* sense RNA interacted with IFIT1 and IFIT3 proteins more than the antisense RNA (Table 2). The IFIT protein family, or IFN-induced protein with tetratricopeptide repeats, includes IFN-stimulated genes (ISGs) that get induced during antiviral immune responses and consist of *IFIT1*, *IFIT2*, *IFIT3*, and *IFIT5* in humans (31). The IFIT proteins are well-known to inhibit translation of both cellular mRNA and viral RNA by either interacting with eukaryotic initiation factor 3 (eIF3) to block translation (32) or by binding directly to the 5′ end of nonself RNAs (33, 34). While IFIT2 and IFIT5 were not identified as *VILMIR*-interacting proteins in the mass spectrometry, the interaction of *VILMIR* sense RNA with IFIT1 and IFIT3 proteins was confirmed by Western blot and densitometry analysis (Fig. 1B). In addition, *VILMIR* sense RNA was found to interact with eight ARSs, which are enzymes responsible for pairing tRNAs with amino acids during translation, as well as ARS-interacting multifunctional protein 1 (AIMP1), which helps form the multi-tRNA synthetase complex (35) (Table 2). Two of these ARSs were confirmed by Western blot and densitometry analysis, QARS1 or glutaminyl-tRNA synthetase 1, and KARS1 or lysyl-tRNA synthetase 1 (Fig. 1C). Therefore, while several nuclear and cytoplasmic protein interactions were confirmed *in vitro* and in the cells, these results suggest that *VILMIR* could function in the nucleus by interacting with proteins such as FUBP1 and PUF60 to regulate transcription or in the cytoplasm by interacting with IFIT1 and IFIT3 and/or QARS1 and KARS1 to regulate translation.

### *VILMIR* overexpression results in minimal fold change differences before interferon-β treatment in A549 epithelial cells

**Fig 2:**
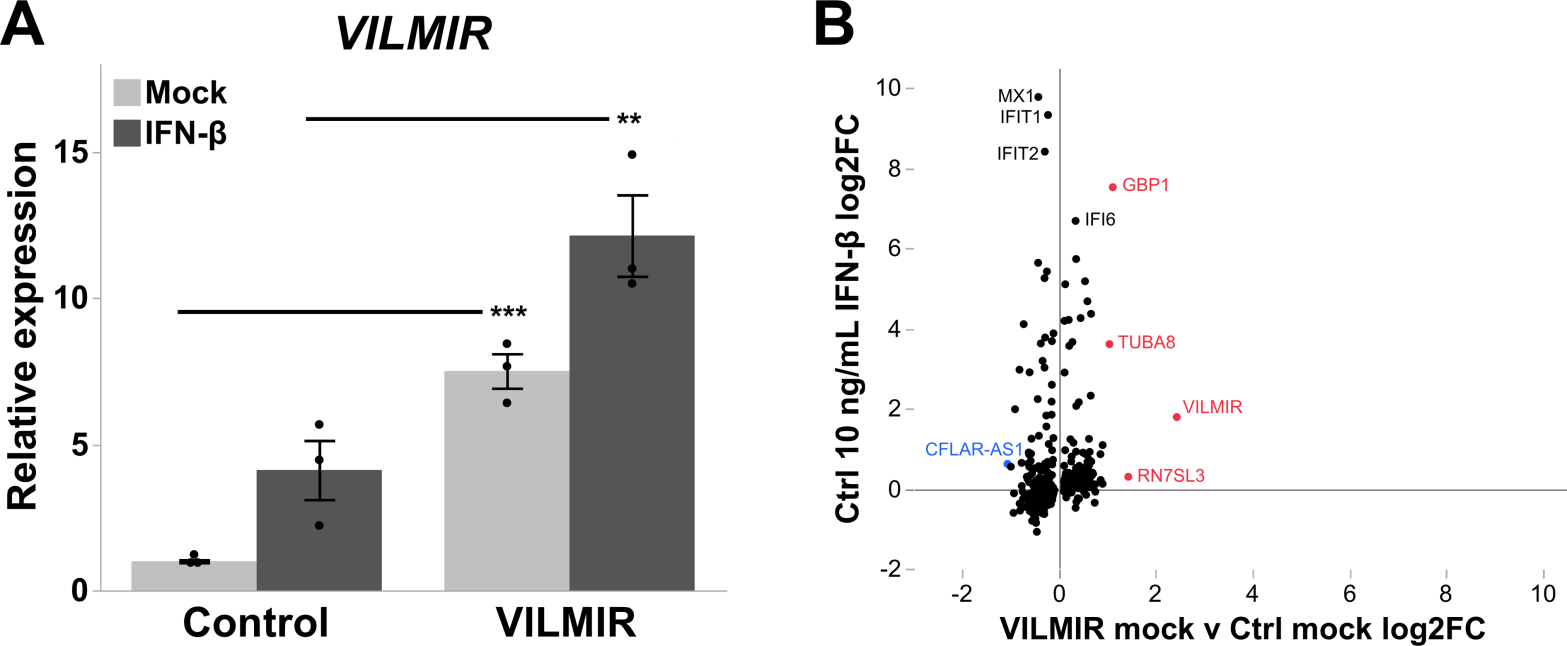
Overexpression of *VILMIR*. (**A**) A549 cells were transduced with a vector expressing ectopic *VILMIR* or an empty-vector control and treated with mock or 10 ng/mL human IFN-β for 6 h. Relative expression of *VILMIR* was determined by RT-qPCR and normalized to the mean of the mock-treated control cell line. Data were normalized to GAPDH using the ΔΔCt method and expressed as means ± SE (*n* = 3). **P < 0.01; ***P < 0.001 (Student’s *t*-test). (**B**) RNA-seq analysis was performed to identify genes that showed altered expression changes after *VILMIR* overexpression in the mock- and IFN-treated cells. Displayed are points representing 459 genes that exhibited significant changes in expression in the *VILMIR*-overexpressing cell line compared to the control cell line in the mock-treated cells (raw *P*-value < 0.05). The x-axis indicates the log2FC difference between each gene in the *VILMIR*-overexpressing cells versus the control cells in the mock (VILMIR mock v Ctrl mock log2FC), while the y-axis represents the log2FC of those same genes after 10 ng/mL IFN-β treatment in the control cell line. The five most differentially expressed genes are labeled in either red (upregulation) or blue (downregulation), as well as four other interferon-stimulated genes (ISGs) of interest in black.

As our pull-down assay suggested that *VILMIR* could function through the transcript itself, we were interested in whether the overexpression of *VILMIR* in cells would cause significant changes in expression, both before and after IFN-β treatment. Therefore, we generated an A549 cell line overexpressing ectopic *VILMIR* and performed RNA-sequencing (RNA-seq) analysis of overexpressing cells treated with or without two separate concentrations of human IFN-β. Compared to the control cell line with an empty vector, we observed a 7.5-fold increase in the baseline expression of *VILMIR* (Fig. 2A). We first examined if *VILMIR* alone caused significant expression differences outside of an IFN response by comparing gene expression in the mock-treated cell lines. Using an adjusted *P*-value < 0.05, the only gene with a significant expression difference in the mock-treated cells after *VILMIR* overexpression was *VILMIR* itself. Therefore, the control cell line and *VILMIR*-overexpressing cell line appeared very similar in gene expressions before IFN-β treatment. When this analysis was expanded to use a relaxed criterion for differential expression analysis (raw *P*-value < 0.05 and no FC cutoff), we identified 459 genes that showed altered expression changes in the *VILMIR*-overexpressing cell line compared to the control cell line in the mock treatment (Fig. 2B).

Interestingly, there were several ISGs impacted by *VILMIR* overexpression in the mock-treated cells, including *MX1*, *IFIT1*, and *IFIT2*, the expressions of which were slightly downregulated, as well as *IFI6*, the expression of which was slightly upregulated (Fig. 2B). This may suggest that *VILMIR* expression regulates ISGs before IFN treatment. However, when plotting these same genes against their log2 fold change (log2FC) after IFN-β treatment in the control cell line, we observed that the log2FC differences between the mock-treated cell lines were relatively small in comparison. In fact, only 14% of the total 459 genes exhibited absolute FC greater than 1.5, and five genes exhibited an absolute FC greater than 2 (Fig. 2B). Apart from *VILMIR* itself, which was the highest upregulated gene as expected, the expressions of *RNS7SL3*, *GBP1*, and *TUBA8* were upregulated and that of *CFLAR-AS1* was downregulated with an FC greater than 2 (Fig. 2B). As *VILMIR* was transcribed ectopically from an overexpression vector, this suggests that *VILMIR* can function through its transcript, rather than just transcription at its genomic locus. In addition, these results suggest that *VILMIR* may have a regulatory role outside of the IFN response, particularly with transcription of *RNS7SL3*, *GBP1*, *TUBA8*, and *CFLAR-AS1*. However, as the majority of expression differences caused by *VILMIR* in the mock-treated cells were relatively small, we sought to determine the impact of *VILMIR* overexpression on host transcription in response to IFN-β treatment.

### Overexpression of *VILMIR* enhances the host transcriptional response to interferon-β treatment in A549 epithelial cells

Next, we determined the impact of *VILMIR* overexpression on the host transcriptional response to IFN-β treatment using the same RNA-seq analysis described above. When using an adjusted *P*-value < 0.05, the only gene that showed an altered expression change to IFN-β treatment after *VILMIR* overexpression was *VILMIR* itself. Therefore, to investigate the potentially broad regulatory roles of *VILMIR*, similarly as in our previous study (19), we used a relaxed criterion for differential expression analysis, i.e., raw *P*-value < 0.05, which allowed us to observe the overall trend of the transcriptional response after *VILMIR* overexpression. Using this criterion, we identified 731 genes that showed altered expression changes to IFN-β treatment after *VILMIR* overexpression in at least one of the two doses of IFN-β (Table S2). When analyzing the host transcriptional response to IFN-β, we observed larger FC differences after *VILMIR* overexpression, with 33%–35% of differentially expressed genes (DEGs) exhibiting absolute FCs greater than 1.5 in either IFN-β treatment, compared to 14% in the mock treatment, indicating that *VILMIR* overexpression has larger impacts during an IFN response. However, similarly to our previous study (19), the magnitude of expression changes was relatively small, with gene expression changing by an average of 1.4-fold after *VILMIR* overexpression compared to the control.

**Fig 3:**
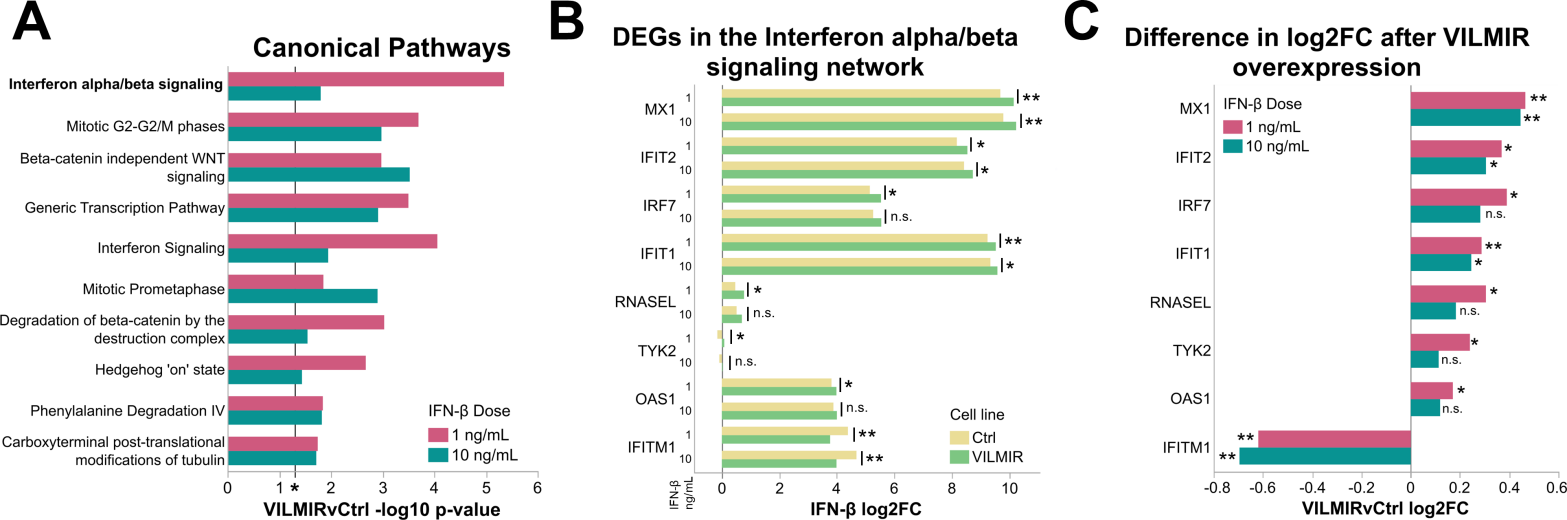
Overexpression of *VILMIR* results in the enhancement of ISGs after IFN-β treatment in A549 cells. (**A**) RNA-seq analysis was performed to identify genes that showed altered expression changes to IFN-β treatment after *VILMIR* overexpression in at least one of two doses of IFN-β (raw *P*-value < 0.05). QIAGEN IPA was then performed to identify canonical pathways significantly enriched in the DEGs impacted by *VILMIR* overexpression after 1 ng/mL or 10 ng/mL IFN-β. Shown are the top 10 out of 17 significant pathways shared between both IFN-β treatments (Table S3). Enriched pathways that met the raw enrichment *P*-value < 0.05 (−log10 *P*-value cutoff of 1.3) using a right-tailed Fisher’s exact test and were associated with a canonical pathway in the QIAGEN Knowledge Base were included here (*P < 0.05 included as the reference). (**B**) Displayed are the DEGs in the interferon alpha/beta signaling network according to IPA, along with their log2FC values after both IFN-β treatments in the Ctrl and VILMIR overexpression cell lines. (**C**) The difference in log2FC of the same genes is shown between VILMIR and Ctrl cell lines (VILMIRvCtrl). *P < 0.05; **P < 0.01.

To identify canonical pathways enriched in the DEGs impacted by *VILMIR* overexpression, QIAGEN IPA was performed (27). There were 17 canonical pathways significantly enriched in the overexpression cell line in both IFN-β treatments with a raw enrichment *P*-value < 0.05 (−log10 *P*-value > 1.3), with the most significant of these pathways being the interferon alpha/beta signaling pathway (Fig. 3A; Table S3). In addition, IPA predicted an overall activation of this pathway, with positive z-scores of 2.121 and 1 for the 1 ng/mL and 10 ng/mL IFN-β treatments, respectively. The majority of the genes represented in this pathway, such as *MX1*, *IFIT2*, *IRF7*, *IFIT1*, *RNASEL*, *TYK2*, and *OAS1,* had higher FCs after *VILMIR* overexpression compared to the control cell line, besides *IFITM1,* which had a lower FC (Fig. 3B and C). However, it is possible that *IFITM1* could serve as a negative regulator as it has previously been found to be a negative regulator in certain contexts, with suppression of *IFITM1* inhibiting proliferation in glioma cells (36). The overall enhancement of genes within the IFN pathway after *VILMIR* overexpression is consistent with our previous findings that *VILMIR* KD suppressed ISGs (19), supporting our hypothesis that *VILMIR* plays an activating role in the host interferon response.

### *VILMIR* knockdown and overexpression consistently regulate the transcription of a core set of interferon-stimulated genes in A549 epithelial cells

**Fig 4:**
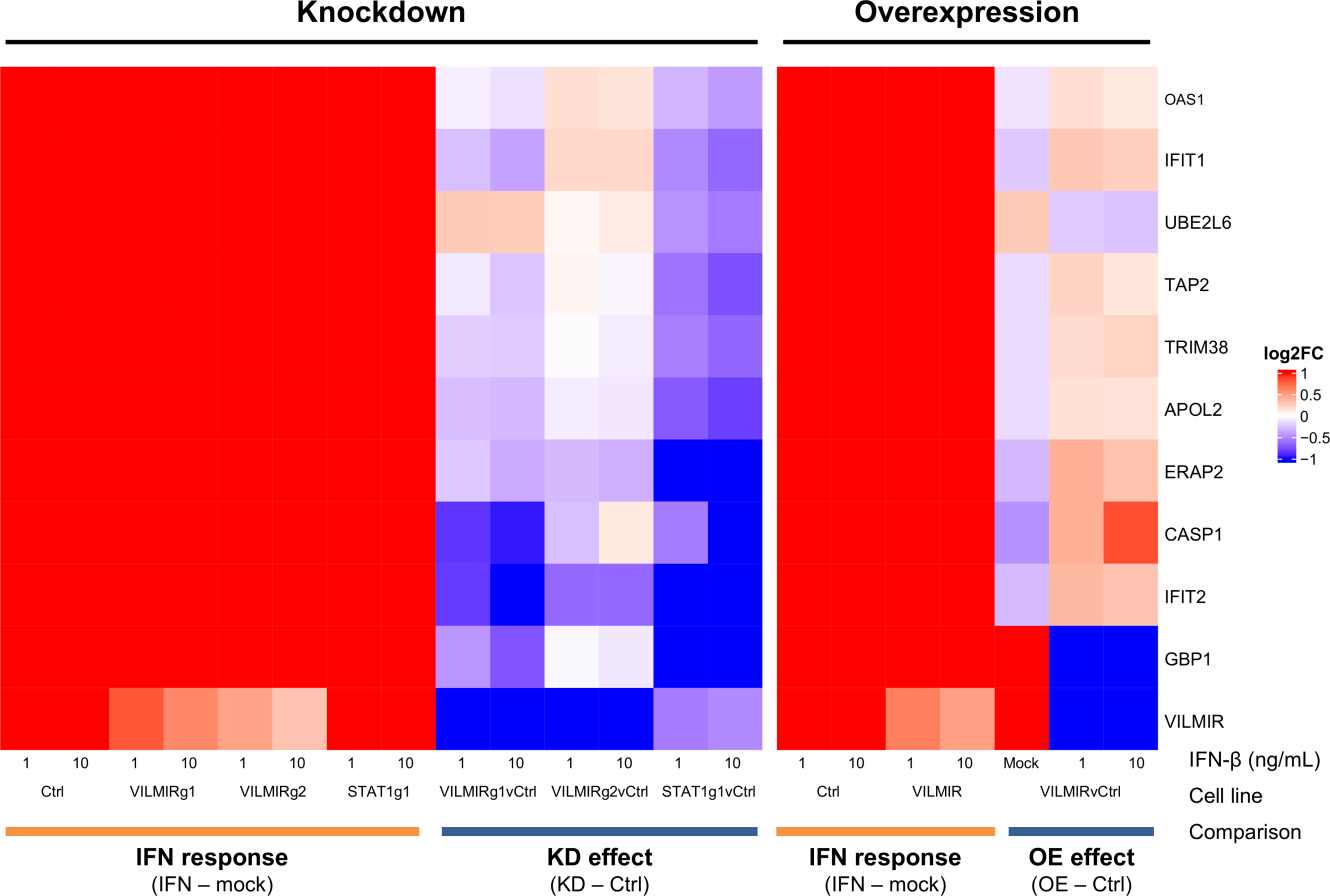
Subset of ISGs that are differentially expressed after *VILMIR* knockdown and overexpression in A549 cells. Heatmap overview of the RNA-seq analysis of two independent experiments: *VILMIR* KD (VILMIRg1 and VILMIRg2), *STAT1* KD (STAT1g1), or control (Ctrl) A549 gRNA cell lines; and *VILMIR* overexpression (VILMIR) or control (Ctrl) A549 cell lines, all of which were treated with mock or either 1 ng/mL or 10 ng/mL human IFN-β for 6 h (*n* = 3). The heatmap displays 11 human genes that exhibited significant changes in their responses to IFN-β treatment after both *VILMIR* KD and overexpression (OE), in at least one of two doses of IFN (raw *P*-value < 0.05). Rows are genes, and columns are conditions and comparisons. As shown by the labels at the bottom, the log2FC after IFN-β treatment in each cell line was first calculated (“IFN response”), and then the “KD effect” or “OE effect” was calculated by comparing the “IFN response” log2FC of each KD/OE line to the “IFN response” log2FC of the control cell line. Red color indicates a positive log2FC value (i.e., upregulation) in columns above the label “IFN response,” or higher log2FC values in KD/OE cells compared to that of control cells in columns above the label “KD effect.” The blue color indicates lower log2FC values in KD/OE cells compared to that of control cells in columns above the label “KD/OE effect.”

In order to identify a core set of genes that were differentially expressed in response to IFN-β treatment after both *VILMIR* overexpression and KD, we next combined the RNA-seq analysis of *VILMIR* overexpression with our previous analysis of *VILMIR* KD (19), which included two A549 *VILMIR* KD cell lines (VILMIRg1 and VILMIRg2) as well as a *STAT1* KD cell line (STAT1g1) as a positive control for interferon response, treated with the same two IFN-β doses. To obtain a more robust set of genes that are IFN-responsive, we applied an additional filtering step of an FC greater than 1.25 after IFN-β treatment in the control cell lines for each experiment. After applying this criterion, we then examined expression changes after *VILMIR* KD or overexpression with a raw *P*-value < 0.05. This gave us a list of 132 IFN-responsive genes that showed altered expression changes to IFN-β treatment after either *VILMIR* KD or overexpression, in at least one of the two IFN-β doses (Fig. S1). Out of these genes, 96 were only differentially expressed after *VILMIR* KD, whereas 25 genes were only differentially expressed after *VILMIR* overexpression. This difference in the number of DEGs may be because the KD study contained two *VILMIR* KD cell lines, whereas the overexpression experiment only had a single *VILMIR* overexpression cell line. Another reason could be due to the difference in the method of gene perturbation as the KD targeted endogenous *VILMIR*, whereas the overexpression produced ectopic *VILMIR* transcripts lacking modifications. The remaining 11 out of 132 genes impacted by *VILMIR* perturbation were differentially expressed after both *VILMIR* KD and overexpression, which is what we chose to focus on (Fig. 4). One of these genes included *VILMIR* itself, which was expected.

The 10 genes that were consistently impacted by *VILMIR* perturbation were *OAS1*, *IFIT1*, *UBE2L6*, *TAP2*, *TRIM38*, *APOL2*, *ERAP2*, *CASP1*, *IFIT2*, and *GBP1*, which have all been associated with interferon and antiviral responses in literature (Table 3). As expected, the expressions of these genes were all upregulated after IFN-β treatment in our A549 cell lines. However, after *VILMIR* KD, we observed an overall suppression of the expression of these genes, whereas after *VILMIR* overexpression, these same genes showed an increase in expression (Fig. 4). The opposite trend was true for *UBE2L6*, which showed an increase in the *VILMIR* KD lines and a decrease in the *VILMIR* overexpression line. However, one reason for this opposite trend may be that *UBE2L6*, a ubiquitin-conjugating enzyme, can be a negative regulator in certain contexts, such as inhibiting autophagy in cancer cells (37). Additionally, *GBP1* was decreased in both *VILMIR* KD and overexpression after IFN-β treatment. However, as *GBP1* was already upregulated in response to *VILMIR* overexpression before IFN-β treatment, it is possible that *VILMIR* upregulates *GBP1* independently of IFN responses (Fig. 4, “Overexpression” mock column, as well as in Fig. 2B), which explains why the addition of IFN in the overexpression cell line results in a smaller FC. This is also true of *VILMIR* itself, which shows a negative FC difference in the IFN-treated overexpression cells. However, because *VILMIR* is already highly upregulated before IFN-β treatment in the overexpression cells, the addition of IFN-β does not result in a higher FC compared to the control cells. Finally, as all 10 genes consistently regulated by *VILMIR* are located on different chromosomes than *VILMIR*, these results suggest that *VILMIR* is a *trans* regulator of gene expression.

**TABLE 3:** Summary of DEGs impacted by both *VILMIR* KD and overexpression, as seen in Fig. 4

Gene	Function	Literature
*OAS1*	Oligoadenylate synthetase 1; recognizes viral dsRNA and activates RNase L degradation and inhibit translation	(38)
*IFIT1*	Binds with eukaryotic initiation factor 3 (eIF3) to block translation; also binds directly to non-self RNAs to inhibit their translation	(32, 34)
*UBE2L6*	Ubiquitin-conjugating enzyme E2 L6; role in protein degradation and ISGylation	(39)
*TAP2*	Transporter associating with antigen processing; transports cytosolic peptides into the ER for presentation by MHC class I molecules	(40)
*TRIM38*	E3 ubiquitin ligase; negatively regulates TLR3-mediated type I IFN signaling by targeting TRIF for degradation	(41)
*APOL2*	Involved in lipid transport and metabolism; also involved in maintaining airway epithelial layer integrity	(42)
*ERAP2*	ER-localized aminopeptidase that plays a role in antigenic peptide editing quality control	(43)
*CASP1*	Cysteine-aspartic acid protease; proteolytically cleaves and activates the pro-inflammatory cytokines IL-1β and IL-18	(44)
*IFIT2*	Binds with eukaryotic initiation factor 3 (eIF3) to block translation; also binds directly to non-self RNAs to inhibit their translation	(32, 34)
*GBP1*	GTPase guanylate-binding protein; forms an antimicrobial coat to capture cytosol-invasive bacteria and pathogen-containing vacuoles	(45)

## DISCUSSION

We previously identified the human lncRNA *VILMIR* as a novel ISG during viral infection and found that KD of *VILMIR* in A549 cells resulted in a suppression of the host transcriptional response to IFN-β treatment and IAV infection. However, the mechanism by which *VILMIR* regulates the host interferon response was not explored. Therefore, in this study, we aimed to identify potential protein interactions as well as gene regulatory networks of *VILMIR* to better understand its molecular interactions and propose models of how it may function during an interferon response.

Using an RNA pull-down assay and mass spectrometry, we found that *VILMIR* RNA interacted with several proteins in nuclear and cytoplasmic lysate from A549 epithelial cells treated with IFN-β. As we previously determined that *VILMIR* is distributed in both the nucleus and the cytoplasm (19), these new results further support that *VILMIR* may function in both compartments through protein interactions. Several lncRNAs have also been found to have dual functions in the nucleus and cytoplasm (18, 46–48). For example, lncRNA HOTAIR can function in the nucleus to regulate gene expression by interacting with histone methyltransferases (46), whereas in the cytoplasm, it can act as a competing endogenous RNA (ceRNA) by interacting with microRNAs (miRNAs) and regulating translation (47). Therefore, it is possible that *VILMIR* could function through different mechanisms in each compartment as well.

Since the KD of *VILMIR* in A549 cells resulted in a suppression of the host transcriptional response to IFN-β treatment and IAV infection, we predicted that *VILMIR* may activate the host IFN-β response (19). Here, we analyzed the impact of *VILMIR* overexpression during IFN-β treatment. Using RNA-seq analysis, we identified 731 genes that showed altered expression changes to IFN-β treatment after *VILMIR* overexpression in at least one of the two doses of IFN-β. These DEGs were enriched for the interferon alpha/beta signaling pathway and displayed an overall enhancement of several ISGs after *VILMIR* overexpression, strongly supporting our hypothesis that *VILMIR* activates the host IFN response.

By combining our *VILMIR* KD and overexpression RNA-seq analysis, we obtained a list of 10 IFN-responsive genes with significant expression changes after both *VILMIR* knockdown and overexpression. Interestingly, seven of the ten genes have known functions related to translational regulation, post-translational regulation by ubiquitination, or protease activity*—OAS1*, *IFIT1*, *UBE2L6*, *TRIM38*, *ERAP2*, *CASP1*, and *IFIT2*. This may mean that *VILMIR* has a regulatory role within translational control or protein processing, which are important cellular processes during a viral infection, as the host translation is tightly regulated in order to limit viral propagation (49). These results were also interesting given our pull-down assay that confirmed the interaction of *VILMIR* with several proteins involved in translation regulation *in vitro*, such as IFIT1, IFIT3, QARS1, and KARS1. Therefore, it is possible *VILMIR* could be regulating ISG expression in the nucleus to modulate their protein abundances, as well as interacting with translational machinery in the cytoplasm. Future work is necessary to determine the potential impact of *VILMIR* on global translation, such as by proteomic analysis or ribosome profiling (50).

### Proposed models of *VILMIR* function

**Fig 5:**
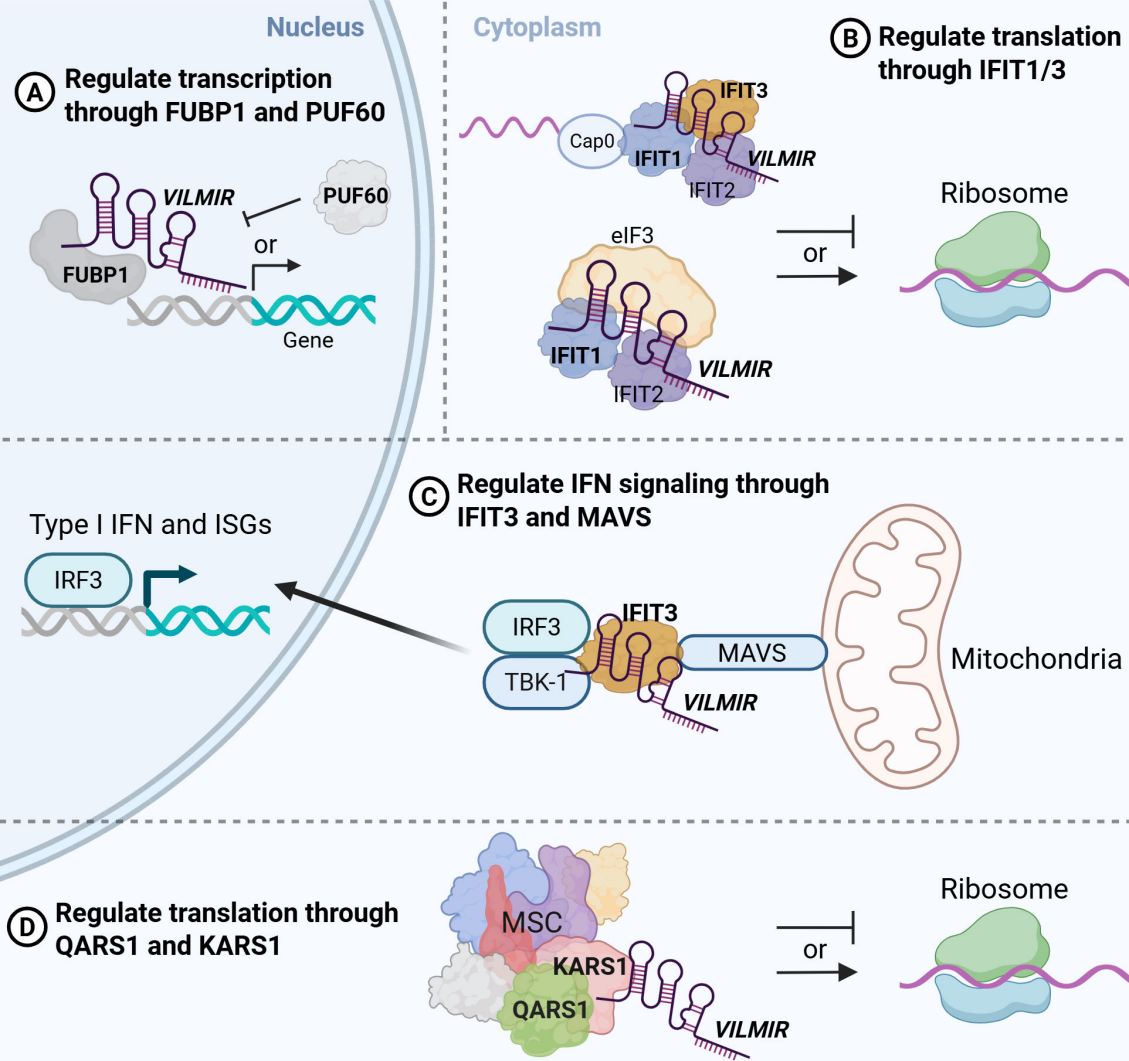
Schematic diagram of potential models of *VILMIR* function in the nucleus and cytoplasm to regulate host interferon responses. (**A**) *VILMIR* may interact with FUBP1 and PUF60 in the nucleus to regulate gene transcription, either by acting as a guide to recruit FUBP1 to enhance transcription or as a decoy to prevent PUF60/FIR from negatively regulating transcription. (**B**) In the cytoplasm, *VILMIR* may interact with IFIT1 and/or IFIT3 to regulate the translation of cellular mRNA and viral RNA, either by the association of IFIT proteins with the 5′ end of RNAs or association with eIF3. (**C**) Additionally, *VILMIR* may act as a scaffold to help bridge the mitochondrial antiviral signaling (MAVS) complex and the TNFR-associated factor family member-associated NF-κB activator-binding kinase 1 (TBK1), which leads to phosphorylation of interferon response factor 3 (IRF3) and induction of IFN-β and ISG expression. (**D**) Finally, *VILMIR* may interact with QARS1 and KARS1 in the MSC to either positively or negatively regulate translation (figure created in https://BioRender.com).

Taking these results together, we suggest several potential models for the function of *VILMIR* that should be further explored. First, as *VILMIR* was found to interact with FUBP1 and PUF60 *in vitro*, known transcriptional regulators in the nucleus (28), we suggest a mechanism by which *VILMIR* interacts with proteins such as FUBP1 and PUF60 to regulate gene transcription in *trans* (Fig. 5A), as we also observed that *VILMIR* KD and overexpression impacts the expression of genes through RNA-seq analysis. FUBP1 has been found to be both a positive regulator of transcription as well as a negative regulator through interacting with the repressor protein FIR or PUF60 (28) and has also been associated with virus infection (29, 51). A different lncRNA, NR-109, was previously found to interact with FUBP1 by preventing ubiquitin-mediated degradation of FUBP1 and thus activating *c-Myc* transcription (52). Therefore, as *VILMIR* overexpression resulted in an activation of ISGs, *VILMIR* may either act as a guide to recruit FUBP1 to enhance transcription or *VILMIR* could act as a decoy to prevent FIR/PUF60 from negatively regulating transcription.

In the cytoplasmic lysate, we confirmed the interaction of *VILMIR* with IFIT1 and IFIT3 *in vitro*. IFIT1 and IFIT3 are known to inhibit translation of both cellular mRNA and viral RNA by either interacting with eIF3 to block translation (32) or by binding directly to the 5′ end of non-self RNAs (33, 34). Therefore, we suggest a second model by which *VILMIR* either stabilizes the functions of IFIT1/3 to inhibit translation or interferes with their function to enhance translation (Fig. 5B). Apart from regulating translation, IFIT3 can also modulate interferon signaling by acting as a bridge between the MAVS complex and the TNFR-associated factor family member-associated NF-κB activator-binding kinase 1 (TBK1), which leads to phosphorylation of IRF3 and induction of IFN-β and ISG expression (53). Therefore, we suggest a third model by which *VILMIR* acts as a scaffold to help bridge IFIT3 to MAVS and TBK1, thus enhancing ISG expression, as we observed that *VILMIR* overexpression resulted in an activation of ISGs (Fig. 5C). Although the IFIT proteins often act in a complex (32), we did not identify IFIT2 or IFIT5 proteins in the mass spectrometry, so it is unknown whether this is due to the sensitivity of the assay or if *VILMIR* does not directly interact with these two proteins. In addition, as IFIT1 and IFIT3 were identified individually by mass spectrometry, the possibility that *VILMIR* interacts with these proteins in a complex needs to be further explored. While previous studies have reported lncRNAs that regulate the transcription of *IFIT* genes (54, 55) and another study reported that a segment of the lncRNA NORAD binds to IFIT proteins (56), to our knowledge, this is the first reported case of a full-length lncRNA interacting with IFIT proteins.

Finally, we also confirmed the interaction of *VILMIR* with two ARSs *in vitro*, QARS1 and KARS1. As stated above, ARSs are enzymes responsible for pairing tRNAs with amino acids during translation and also help form the MSC (35). A study in 2020 found that mascRNA, a small RNA derived from the lncRNA *MALAT1*, binds to QARS1 in the MSC in order to promote global protein translation by regulating QARS1 protein levels (57). Similarly, we suggest a final model by which *VILMIR* either stabilizes QARS1 and/or KARS1 in the MSC to promote translation or interferes with their function to negatively regulate translation (Fig. 5D). While these potential models need to be investigated further, they suggest that *VILMIR* may regulate host interferon responses through protein partners in both the nucleus and cytoplasm, which provides important foundational work in interrogating the specific mechanism of *VILMIR*.

As the pull-down assay was performed *in vitro,* future work is needed to confirm these protein interactions in cells, such as by an RNA immunoprecipitation (RIP) or RNA antisense purification (RAP) assay to establish their biological significance (58). Additional interacting molecules of *VILMIR* may also be determined by assays such as chromatin isolation by RNA purification (ChIRP) that can identify both chromatin and protein associations (58). This could also help determine if *VILMIR* is directly regulating the transcription of specific genes. In addition, immunoprecipitation assays could determine if *VILMIR* was binding to a unique protein or interacting with a protein complex. Finally, the biological significance of these protein interactions could be investigated by blocking or mutating the binding site on either *VILMIR* or the protein, such as in previous studies (14, 59).

In summary, we found that *VILMIR* interacts with multiple proteins in nuclear and cytoplasmic lysate. We also confirmed that *VILMIR* plays an activating role in the host interferon response in *trans* through the establishment of an overexpression cell line. Finally, by compiling RNA-seq analyses, we identified a core set of genes that are consistently differentially expressed after both *VILMIR* KD and overexpression. We have proposed several potential models for how *VILMIR* may function in the host interferon response. We expect these results will serve as a guide in probing the molecular mechanisms of *VILMIR* in detail, providing new insights into the biological significance of *VILMIR* during antiviral and interferon responses.

## Data Availability

The transcriptomic data discussed in this publication have been deposited in the Gene Expression Omnibus (GEO, https://www.ncbi.nlm.nih.gov/geo/) under accession numbers GSE261920 and GSE305270.

## SUPPLEMENTAL MATERIAL

Supplemental material jvi.01380-25-s0001.docx Tables S1 and S3; Fig. S1.

Table S2 jvi.01380-25-s0002.xlsx RNA-seq analysis of 731 genes impacted by VILMIR overexpression after 1 ng/mL or 10 ng/mL IFN-β treatment in A549 cells.

ASM does not own the copyrights to Supplemental Material that may be linked to, or accessed through, an article. The authors have granted ASM a non-exclusive, world-wide license to publish the Supplemental Material files. Please contact the corresponding author directly for reuse.

## References

[1] Catalanotto C, Barbato C, Cogoni C, Benelli D. 2023. The RNA-binding function of ribosomal proteins and ribosome biogenesis factors in human health and disease. Biomedicines 11:2969. doi:10.3390/biomedicines11112969 38001969 PMC10669870

[2] Schuntermann DB, Jaskolowski M, Reynolds NM, Vargas-Rodriguez O. 2024. The central role of transfer RNAs in mistranslation. J Biol Chem 300:107679. doi:10.1016/j.jbc.2024.107679 39154912 PMC11415595

[3] Valadkhan S, Gunawardane LS. 2013. Role of small nuclear RNAs in eukaryotic gene expression. Essays Biochem 54:79–90. doi:10.1042/bse0540079 23829528 PMC11246792

[4] Huang ZH, Du YP, Wen JT, Lu BF, Zhao Y. 2022. snoRNAs: functions and mechanisms in biological processes, and roles in tumor pathophysiology. Cell Death Discov 8:259. doi:10.1038/s41420-022-01056-8 35552378 PMC9098889

[5] Mattick JS, Amaral PP, Carninci P, Carpenter S, Chang HY, Chen L-L, Chen R, Dean C, Dinger ME, Fitzgerald KA, et al.. 2023. Long non-coding RNAs: definitions, functions, challenges and recommendations. Nat Rev Mol Cell Biol 24:430–447. doi:10.1038/s41580-022-00566-8 36596869 PMC10213152

[6] Mudge JM, Carbonell-Sala S, Diekhans M, Martinez JG, Hunt T, Jungreis I, Loveland JE, Arnan C, Barnes I, Bennett R, et al.. 2025. GENCODE 2025: reference gene annotation for human and mouse. Nucleic Acids Res 53:D966–D975. doi:10.1093/nar/gkae1078 39565199 PMC11701607

[7] Flynn RA, Chang HY. 2014. Long noncoding RNAs in cell-fate programming and reprogramming. Cell Stem Cell 14:752–761. doi:10.1016/j.stem.2014.05.014 24905165 PMC4120821

[8] Ma Y, Zhang J, Wen L, Lin A. 2018. Membrane-lipid associated lncRNA: a new regulator in cancer signaling. Cancer Lett 419:27–29. doi:10.1016/j.canlet.2018.01.008 29330108

[9] Atianand MK, Hu W, Satpathy AT, Shen Y, Ricci EP, Alvarez-Dominguez JR, Bhatta A, Schattgen SA, McGowan JD, Blin J, Braun JE, Gandhi P, Moore MJ, Chang HY, Lodish HF, Caffrey DR, Fitzgerald KA. 2016. A long noncoding RNA lincRNA-EPS acts as a transcriptional brake to restrain inflammation. Cell 165:1672–1685. doi:10.1016/j.cell.2016.05.075 27315481 PMC5289747

[10] Jarroux J, Morillon A, Pinskaya M. 2017. History, discovery, and classification of lncRNAs. Adv Exp Med Biol 1008:1–46. doi:10.1007/978-981-10-5203-3_1 28815535

[11] Ginn L, La Montagna M, Wu Q, Shi L. 2021. Diverse roles of long non‐coding RNAs in viral diseases. Rev Med Virol 31. doi:10.1002/rmv.2198

[12] Hu J, Zhang L, Zheng X, Wang G, Chen X, Hu Z, Chen Y, Wang X, Gu M, Hu S, Liu X, Jiao X, Peng D, Liu X. 2023. Long noncoding RNA #61 exerts a broad anti-influenza a virus effect by its long arm rings. Antiviral Res 215:105637. doi:10.1016/j.antiviral.2023.105637 37196902

[13] Carpenter S, Aiello D, Atianand MK, Ricci EP, Gandhi P, Hall LL, Byron M, Monks B, Henry-Bezy M, Lawrence JB, O’Neill LAJ, Moore MJ, Caffrey DR, Fitzgerald KA. 2013. A long noncoding RNA mediates both activation and repression of immune response genes. Science 341:789–792. doi:10.1126/science.1240925 23907535 PMC4376668

[14] Yoon J-H, Abdelmohsen K, Kim J, Yang X, Martindale JL, Tominaga-Yamanaka K, White EJ, Orjalo AV, Rinn JL, Kreft SG, Wilson GM, Gorospe M. 2013. Scaffold function of long non-coding RNA HOTAIR in protein ubiquitination. Nat Commun 4:2939–2939. doi:10.1038/ncomms3939 24326307 PMC4556280

[15] Engreitz JM, Haines JE, Perez EM, Munson G, Chen J, Kane M, McDonel PE, Guttman M, Lander ES. 2016. Local regulation of gene expression by lncRNA promoters, transcription and splicing. Nature 539:452–455. doi:10.1038/nature20149 27783602 PMC6853796

[16] Yan P, Luo S, Lu JY, Shen X. 2017. Cis- and trans-acting lncRNAs in pluripotency and reprogramming. Curr Opin Genet Dev 46:170–178. doi:10.1016/j.gde.2017.07.009 28843809

[17] Elling R, Robinson EK, Shapleigh B, Liapis SC, Covarrubias S, Katzman S, Groff AF, Jiang Z, Agarwal S, Motwani M, Chan J, Sharma S, Hennessy EJ, FitzGerald GA, McManus MT, Rinn JL, Fitzgerald KA, Carpenter S. 2018. Genetic models reveal cis and trans immune-regulatory activities for lincRNA-Cox2. Cell Rep 25:1511–1524. doi:10.1016/j.celrep.2018.10.027 30404006 PMC6291222

[18] Miao H, Wang L, Zhan H, Dai J, Chang Y, Wu F, Liu T, Liu Z, Gao C, Li L, Song X. 2019. A long noncoding RNA distributed in both nucleus and cytoplasm operates in the PYCARD-regulated apoptosis by coordinating the epigenetic and translational regulation. PLoS Genet 15:e1008144. doi:10.1371/journal.pgen.1008144 31086376 PMC6534332

[19] John K, Huntress I, Smith E, Chou H, Tollison TS, Covarrubias S, Crisci E, Carpenter S, Peng X. 2025. Human long noncoding RNA VILMIR is induced by major respiratory viral infections and modulates the host interferon response. J Virol 99:e0014125. doi:10.1128/jvi.00141-25 40130878 PMC11998520

[20] Chaparian RR, van Kessel JC. 2021. Promoter pull-down assay: a biochemical screen for DNA-binding proteins. Methods Mol Biol 2346:165–172. doi:10.1007/7651_2020_307 32803537

[21] Winterling C, Koch M, Koeppel M, Garcia-Alcalde F, Karlas A, Meyer TF. 2014. Evidence for a crucial role of a host non-coding RNA in influenza A virus replication. RNA Biol 11:66–75. doi:10.4161/rna.27504 24440876 PMC3929426

[22] Vollmers AC, Covarrubias S, Kuang D, Shulkin A, Iwuagwu J, Katzman S, Song R, Viswanathan K, Vollmers C, Wakeland E, Carpenter S. 2021. A conserved long noncoding RNA, GAPLINC, modulates the immune response during endotoxic shock. Proc Natl Acad Sci USA 118:e2016648118. doi:10.1073/pnas.2016648118 33568531 PMC7896317

[23] Dobin A, Davis CA, Schlesinger F, Drenkow J, Zaleski C, Jha S, Batut P, Chaisson M, Gingeras TR. 2013. STAR: ultrafast universal RNA-seq aligner. Bioinformatics 29:15–21. doi:10.1093/bioinformatics/bts635 23104886 PMC3530905

[24] Robinson MD, McCarthy DJ, Smyth GK. 2010. edgeR: a Bioconductor package for differential expression analysis of digital gene expression data. Bioinformatics 26:139–140. doi:10.1093/bioinformatics/btp616 19910308 PMC2796818

[25] Ritchie ME, Phipson B, Wu D, Hu Y, Law CW, Shi W, Smyth GK. 2015. Limma powers differential expression analyses for RNA-sequencing and microarray studies. Nucleic Acids Res 43:e47. doi:10.1093/nar/gkv007 25605792 PMC4402510

[26] Gu Z, Eils R, Schlesner M. 2016. Complex heatmaps reveal patterns and correlations in multidimensional genomic data. Bioinformatics 32:2847–2849. doi:10.1093/bioinformatics/btw313 27207943

[27] Krämer A, Green J, Pollard JJ, Tugendreich S. 2014. Causal analysis approaches in ingenuity pathway analysis. Bioinformatics 30:523–530. doi:10.1093/bioinformatics/btt703 24336805 PMC3928520

[28] Debaize L, Troadec M-B. 2019. The master regulator FUBP1: its emerging role in normal cell function and malignant development. Cell Mol Life Sci 76:259–281. doi:10.1007/s00018-018-2933-6 30343319 PMC11105487

[29] Chien H-L, Liao C-L, Lin Y-L. 2011. FUSE binding protein 1 interacts with untranslated regions of Japanese encephalitis virus RNA and negatively regulates viral replication. J Virol 85:4698–4706. doi:10.1128/JVI.01950-10 21367899 PMC3126168

[30] Jacob AG, Singh RK, Mohammad F, Bebee TW, Chandler DS. 2014. The splicing factor FUBP1 is required for the efficient splicing of oncogene MDM2 pre-mRNA. J Biol Chem 289:17350–17364. doi:10.1074/jbc.M114.554717 24798327 PMC4067169

[31] Diamond MS, Farzan M. 2013. The broad-spectrum antiviral functions of IFIT and IFITM proteins. Nat Rev Immunol 13:46–57. doi:10.1038/nri3344 23237964 PMC3773942

[32] Mears HV, Sweeney TR. 2018. Better together: the role of IFIT protein-protein interactions in the antiviral response. J Gen Virol 99:1463–1477. doi:10.1099/jgv.0.001149 30234477

[33] Pichlmair A, Lassnig C, Eberle C-A, Górna MW, Baumann CL, Burkard TR, Bürckstümmer T, Stefanovic A, Krieger S, Bennett KL, Rülicke T, Weber F, Colinge J, Müller M, Superti-Furga G. 2011. IFIT1 is an antiviral protein that recognizes 5’-triphosphate RNA. Nat Immunol 12:624–630. doi:10.1038/ni.2048 21642987

[34] Kumar P, Sweeney TR, Skabkin MA, Skabkina OV, Hellen CUT, Pestova TV. 2014. Inhibition of translation by IFIT family members is determined by their ability to interact selectively with the 5’-terminal regions of cap0-, cap1- and 5’ppp- mRNAs. Nucleic Acids Res 42:3228–3245. doi:10.1093/nar/gkt1321 24371270 PMC3950709

[35] Rajendran V, Kalita P, Shukla H, Kumar A, Tripathi T. 2018. Aminoacyl-tRNA synthetases: structure, function, and drug discovery. Int J Biol Macromol 111:400–414. doi:10.1016/j.ijbiomac.2017.12.157 29305884

[36] Yu F, Ng SSM, Chow BKC, Sze J, Lu G, Poon WS, Kung H-F, Lin MCM. 2011. Knockdown of interferon-induced transmembrane protein 1 (IFITM1) inhibits proliferation, migration, and invasion of glioma cells. J Neurooncol 103:187–195. doi:10.1007/s11060-010-0377-4 20838853 PMC3097340

[37] Falvey CM, O’Donovan TR, El-Mashed S, Nyhan MJ, O’Reilly S, McKenna SL. 2017. UBE2L6/UBCH8 and ISG15 attenuate autophagy in esophageal cancer cells. Oncotarget 8:23479–23491. doi:10.18632/oncotarget.15182 28186990 PMC5410320

[38] Harioudh MK, Perez J, Chong Z, Nair S, So L, McCormick KD, Ghosh A, Shao L, Srivastava R, Soveg F, Ebert TS, Atianand MK, Hornung V, Savan R, Diamond MS, Sarkar SN. 2024. Oligoadenylate synthetase 1 displays dual antiviral mechanisms in driving translational shutdown and protecting interferon production. Immunity 57:446–461. doi:10.1016/j.immuni.2024.02.002 38423012 PMC10939734

[39] Zhang D, Zhang DE. 2011. Interferon-stimulated gene 15 and the protein ISGylation system. J Interferon Cytokine Res 31:119–130. doi:10.1089/jir.2010.0110 21190487 PMC3021351

[40] Mantel I, Sadiq BA, Blander JM. 2022. Spotlight on TAP and its vital role in antigen presentation and cross-presentation. Mol Immunol 142:105–119. doi:10.1016/j.molimm.2021.12.013 34973498 PMC9241385

[41] Xue Q, Zhou Z, Lei X, Liu X, He B, Wang J, Hung T. 2012. TRIM38 negatively regulates TLR3-mediated IFN-β signaling by targeting TRIF for degradation. PLoS One 7:e46825. doi:10.1371/journal.pone.0046825 23056470 PMC3466175

[42] Liao W, Goh FY, Betts RJ, Kemeny DM, Tam J, Bay B-H, Wong WSF. 2011. A novel anti-apoptotic role for apolipoprotein L2 in IFN-γ-induced cytotoxicity in human bronchial epithelial cells. J Cell Physiol 226:397–406. doi:10.1002/jcp.22345 20665705

[43] Saulle I, Marventano I, Saresella M, Vanetti C, Garziano M, Fenizia C, Trabattoni D, Clerici M, Biasin M. 2021. ERAPs reduce in vitro HIV infection by activating innate immune response. J Immunol 206:1609–1617. doi:10.4049/jimmunol.2000991 33619214 PMC7980528

[44] Bauer RN, Brighton LE, Mueller L, Xiang Z, Rager JE, Fry RC, Peden DB, Jaspers I. 2012. Influenza enhances caspase-1 in bronchial epithelial cells from asthmatic volunteers and is associated with pathogenesis. J Allergy Clin Immunol 130:958–67. doi:10.1016/j.jaci.2012.07.013 23021143 PMC3470476

[45] Anonymous. 2025. The guanylate-binding protein GBP1 forms a protein coat that enwraps cytosol-invasive bacteria. Nat Struct Mol Biol 32:8–9. doi:10.1038/s41594-024-01403-6 39406982

[46] Bhan A, Mandal SS. 2015. LncRNA HOTAIR: a master regulator of chromatin dynamics and cancer. Biochim Biophys Acta 1856:151–164. doi:10.1016/j.bbcan.2015.07.001 26208723 PMC4544839

[47] Li Y, Sun W, Li J, Du R, Xing W, Yuan X, Zhong G, Zhao D, Liu Z, Jin X, Pan J, Li Y, Li Q, Kan G, Han X, Ling S, Sun X, Li Y. 2023. HuR-mediated nucleocytoplasmic translocation of HOTAIR relieves its inhibition of osteogenic differentiation and promotes bone formation. Bone Res 11:53. doi:10.1038/s41413-023-00289-2 37872163 PMC10593784

[48] Katsushima K, Natsume A, Ohka F, Shinjo K, Hatanaka A, Ichimura N, Sato S, Takahashi S, Kimura H, Totoki Y, Shibata T, Naito M, Kim HJ, Miyata K, Kataoka K, Kondo Y. 2016. Targeting the Notch-regulated non-coding RNA TUG1 for glioma treatment. Nat Commun 7:13616. doi:10.1038/ncomms13616 27922002 PMC5150648

[49] Hoang HD, Neault S, Pelin A, Alain T. 2021. Emerging translation strategies during virus-host interaction. Wiley Interdiscip Rev RNA 12:e1619. doi:10.1002/wrna.1619 32757266 PMC7435527

[50] Brar GA, Weissman JS. 2015. Ribosome profiling reveals the what, when, where and how of protein synthesis. Nat Rev Mol Cell Biol 16:651–664. doi:10.1038/nrm4069 26465719 PMC5522010

[51] Dixit U, Pandey AK, Liu Z, Kumar S, Neiditch MB, Klein KM, Pandey VN. 2015. FUSE binding protein 1 facilitates persistent hepatitis C virus replication in hepatoma cells by regulating tumor suppressor p53. J Virol 89:7905–7921. doi:10.1128/JVI.00729-15 25995247 PMC4505638

[52] Zhang C, Wei S, Dai S, Li X, Wang H, Zhang H, Sun G, Shan B, Zhao L. 2023. The NR_109/FUBP1/c-Myc axis regulates TAM polarization and remodels the tumor microenvironment to promote cancer development. J Immunother Cancer 11:e006230. doi:10.1136/jitc-2022-006230 37217247 PMC10230994

[53] Liu X-Y, Chen W, Wei B, Shan Y-F, Wang C. 2011. IFN-induced TPR protein IFIT3 potentiates antiviral signaling by bridging MAVS and TBK1. J Immunol 187:2559–2568. doi:10.4049/jimmunol.1100963 21813773

[54] Guo B, Jiang T, Wu F, Ni H, Ye J, Wu X, Ni C, Jiang M, Ye L, Li Z, Zheng X, Li S, Yang Q, Wang Z, Huang X, Zhao C. 2022. LncRNA RP5-998N21.4 promotes immune defense through upregulation of IFIT2 and IFIT3 in schizophrenia. Schizophrenia (Heidelb) 8:11. doi:10.1038/s41537-021-00195-8 35232977 PMC8888552

[55] van Solingen C, Cyr Y, Scacalossi KR, de Vries M, Barrett TJ, de Jong A, Gourvest M, Zhang T, Peled D, Kher R, Cornwell M, Gildea MA, Brown EJ, Fanucchi S, Mhlanga MM, Berger JS, Dittmann M, Moore KJ. 2022. Long noncoding RNA CHROMR regulates antiviral immunity in humans. Proc Natl Acad Sci USA 119:e2210321119. doi:10.1073/pnas.2210321119 36001732 PMC9477407

[56] Tichon A, Gil N, Lubelsky Y, Havkin Solomon T, Lemze D, Itzkovitz S, Stern-Ginossar N, Ulitsky I. 2016. A conserved abundant cytoplasmic long noncoding RNA modulates repression by Pumilio proteins in human cells. Nat Commun 7:12209. doi:10.1038/ncomms12209 27406171 PMC4947167

[57] Lu X, Huang J, Wu S, Zheng Q, Liu P, Feng H, Su X, Fu H, Xi Q, Wang G. 2020. The tRNA-like small noncoding RNA mascRNA promotes global protein translation. EMBO Rep 21:e49684. doi:10.15252/embr.201949684 33073493 PMC7726780

[58] Wang H-L, Chekanova JA. 2019. An overview of methodologies in studying lncRNAs in the high-throughput era: when acronyms ATTACK! Methods Mol Biol 1933:1–30. doi:10.1007/978-1-4939-9045-0_1 30945176 PMC6684206

[59] Hu G, Lou Z, Gupta M. 2014. The long non-coding RNA GAS5 cooperates with the eukaryotic translation initiation factor 4E to regulate c-Myc translation. PLoS One 9:e107016. doi:10.1371/journal.pone.0107016 25197831 PMC4157848

