# Supplementary material for: VILMIR is a trans-acting long noncoding RNA that enhances the host interferon response in human epithelial cells
Source: J Virol. 2025 Dec 11;100(1):e01380-25. doi: 10.1128/jvi.01380-25 (PMC12817941; doi:10.1128/jvi.01380-25)
Supplement: Supplemental material — Tables S1 and S3; Fig. S1. [file jvi.01380-25-s0001.docx]

**Supplementary MATERIALS**

**Table S1.** Full-length sequence of *VILMIR*.

GGGGCTCCGGGAGTCACCCCCAGACGCGCGGCTCCCCTTCCTTGTCCTCCGCATGACGCCCGTGCCGGAGCCCACGTGAGCCTTTGCCAAGGAGAGATTCAGAGATATGAGCAGAAAAACTGGAAGATGGACTTCTTCAATAAGGCAGCGGGACGGTTTGGCTGATGGAAGATGTTTCCCGGAAGCCTGTTCACGCTCCACCCTGAAAGTCCCAGGGCCGTGAGAGTCAGCCGGGCCGCCGAGGCGGTGCCCTCCAGGCTGCAGGATGATCCAGGGACTGTTTTCCTCAGCACTGTGTAGGTTTCAGCTTATGAGTTCTGTCCATGTTTTGTGAGATTTATAGCTAAGTATTTACCTTTTCGAATGATTATAAACAACATAGTAGTTTTAATTTTAGTGTCTACTTGTTTTTTGTTACTATATAGAAATACAATCGATTTTCATATATTTATGTTGCATCCTCTGTGCTTCTAAACTCACTAGTTTTGGAAGCTGTTTTTTGTTTTTAGATTCCTTAAAATTTTATATATGGGCAATCATGTCATTTAATAATCAACCTGTATGCCTAAAATAATCAACCTGTATGCCTAAAATAATCAACCTGTATGCCTTTTATTGTAAGAAAATCCCTAAATACTGGGATTACAGGTGGTGAGCCACCGTGTCCAACCTGAAACTTTTTTTTTGGTGATTTCAAATGAACTTGACTTTTACTGTAATTATACCTAAAGTATTTGTAAACAATTGTTTAGAACTTCTATTTGTCATGGACTTTTGAGTTTATTCTTTGGATCATATAAAAAGACTTTTTTTTTTTGTTTTTAAAGTTATAAATCTCTGTATGCACTTTGCTTTTCTTAATTAAACATAATCCAAA

**Table S3.** Canonical pathways enriched in *VILMIR* overexpression after two IFN-β treatments in A549 cells as determined by Ingenuity Pathway Analysis, shown in Figure 3.

| **Canonical Pathways** | **-log10 p-value** | |
| --- | --- | --- |
|  | **VILMIRvCtrl 1 ng IFN** | **VILMIRvCtrl 10 ng IFN** |
| Interferon alpha/beta signaling | 5.350204 | 1.802966 |
| Mitotic G2-G2/M phases | 3.694762 | 2.978204 |
| Beta-catenin independent WNT signaling | 2.975993 | 3.525354 |
| Generic Transcription Pathway | 3.500226 | 2.913881 |
| Interferon Signaling | 4.059731 | 1.947017 |
| Mitotic Prometaphase | 1.854533 | 2.903417 |
| Degradation of beta-catenin by the destruction complex | 3.031983 | 1.547394 |
| Hedgehog 'on' state | 2.67427 | 1.439774 |
| Phenylalanine Degradation IV (Mammalian, via Side Chain) | 1.844238 | 1.824403 |
| Carboxyterminal post-translational modifications of tubulin | 1.744904 | 1.717457 |
| Deubiquitination | 1.75586 | 1.700174 |
| Heme signaling | 1.694756 | 1.667495 |
| PTEN Regulation | 1.413294 | 1.91377 |
| Transcriptional regulation by RUNX3 | 1.50663 | 1.473893 |
| Neddylation | 1.474352 | 1.426699 |
| Circadian Clock | 1.457973 | 1.431729 |
| Cilium Assembly | 1.383352 | 1.341347 |

**
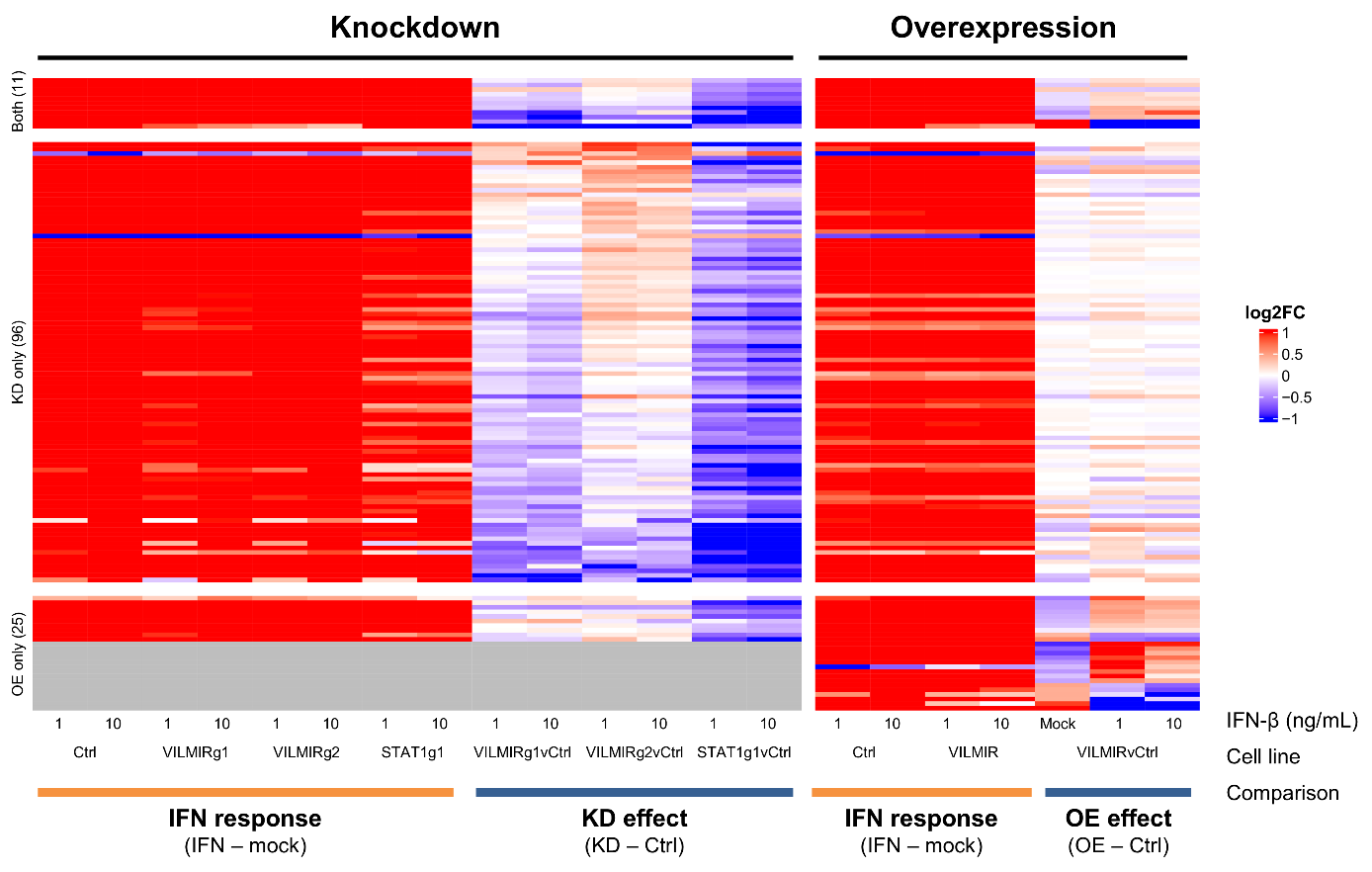
**

**Figure S1. Interferon-stimulated genes that are differentially expressed after *VILMIR* knockdown and overexpression in A549 cells.** Heatmap overview of the RNA-seq analysis of two independent experiments: *VILMIR* knockdown (VILMIRg1 and VILMIRg2), *STAT1* knockdown (STAT1g1), or control (Ctrl) A549 gRNA cell lines; and *VILMIR* overexpression (VILMIR) or control (Ctrl) A549 cell lines; all of which were treated with mock or either 1 ng/mL or 10 ng/mL human IFN-β for 6 hours (n = 3). The heatmap displays 132 human genes that exhibited significant changes in their responses to IFN-β treatment after either *VILMIR* KD or overexpression (OE), in at least one of two doses of IFN (raw P-value <0.05). Rows are genes and columns are conditions and comparisons. As shown by the labels at the bottom, the log2FC after IFN-β treatment in each cell line was first calculated (“IFN response”), and then the “KD effect” or “OE effect” was calculated by comparing the “IFN response” log2FC of each KD/OE line to the “IFN response” log2FC of the control cell line. Red color indicates positive log2FC value (i.e., upregulation) in columns above the label “IFN response,” or higher log2FC values in KD/OE cells compared to that of control cells in columns above the label “KD/OE effect.” The blue color indicates lower log2FC values in KD/OE cells compared to that of control cells in columns above the label “KD/OE effect.”
